# The effect of audit and feedback and implementation support on guideline adherence and patient outcomes in cardiac rehabilitation: a study protocol for an open-label cluster-randomized effectiveness-implementation hybrid trial

**DOI:** 10.1186/s13012-024-01366-8

**Published:** 2024-05-24

**Authors:** Halldóra Ögmundsdóttir Michelsen, Matthias Lidin, Maria Bäck, Therese Scott Duncan, Björn Ekman, Emil Hagström, Maria Hägglund, Bertil Lindahl, Mona Schlyter, Margrét Leósdóttir

**Affiliations:** 1 Department of Clinical Sciences Malmö, Lund University, Malmö, Sweden; 2 Department of Emergency medicine and Geriatrics, Helsingborg Hospital, Helsingborg, Sweden; 3 Department of Medicine, Karolinska Institute, Solna, Stockholm, Sweden; 4 Department of Cardiology, Heart, Vascular and Neuro Theme, Karolinska University Hospital, Stockholm, Sweden; 5 Department of Occupational Therapy and Physiotherapy, Sahlgrenska University Hospital, Gothenburg, Sweden; 6 Department of Health, Medicine and Caring Sciences, Unit of Physiotherapy, Linköping University, Linköping, Sweden; 7 Department of Women’s and Children’s Health, Uppsala University, Uppsala, Sweden; 8 Department of Medical Sciences, Cardiology, Uppsala University, Uppsala, Sweden; 9 Uppsala Clinical Research Centre, Uppsala, Sweden; 10 Department of Cardiology, Skåne University Hospital, Malmö, Sweden

**Keywords:** Cardiac rehabilitation, Cost-effectiveness, Guidelines, Implementation support, Myocardial infarction, Registry, Secondary prevention

## Abstract

**Background:**

Providing secondary prevention through structured and comprehensive cardiac rehabilitation programmes to patients after a myocardial infarction (MI) reduces mortality and morbidity and improves health-related quality of life. Cardiac rehabilitation has the highest recommendation in current guidelines. While treatment target attainment rates at Swedish cardiac rehabilitation centres is among the highest in Europe, there are considerable differences in service delivery and variations in patient-level outcomes between centres. In this trial, we aim to study whether centre-level guideline adherence and patient-level outcomes across Swedish cardiac rehabilitation centres can be improved through a) regular audit and feedback of cardiac rehabilitation structure and processes through a national quality registry and b) supporting cardiac rehabilitation centres in implementing guidelines on secondary prevention. Furthermore, we aim to evaluate the implementation process and costs.

**Methods:**

The study is an open-label cluster-randomized effectiveness-implementation hybrid trial including all 78 cardiac rehabilitation centres (attending to approximately 10 000 MI patients/year) that report to the SWEDEHEART registry. The centres will be randomized 1:1:1 to three clusters: 1) reporting cardiac rehabilitation structure and process variables to SWEDEHEART every six months (audit intervention) and being offered implementation support to implement guidelines on secondary prevention (implementation support intervention); 2) audit intervention only; or 3) no intervention offered. Baseline cardiac rehabilitation structure and process variables will be collected. The primary outcome is an adherence score measuring centre-level adherence to secondary prevention guidelines. Secondary outcomes include patient-level secondary prevention risk factor goal attainment at one-year after MI and major adverse coronary outcomes for up to five-years post-MI. Implementation outcomes include barriers and facilitators to guideline adherence evaluated using semi-structured focus-group interviews and relevant questionnaires, as well as costs and cost-effectiveness assessed by a comparative health economic evaluation.

**Discussion:**

Optimizing cardiac rehabilitation centres’ delivery of services to meet standards set in guidelines may lead to improvement in cardiovascular risk factors, including lifestyle factors, and ultimately a decrease in morbidity and mortality after MI.

**Trial registration:**

ClinicalTrials.gov. Identifier: NCT05889416. Registered 2023-03-23.

**Supplementary Information:**

The online version contains supplementary material available at 10.1186/s13012-024-01366-8.

Contributions to the literature

- Structured implementation support to improve guideline adherence has not previously been assessed within cardiac rehabilitation.
- The study is a national open-label cluster-randomized effectiveness-implementation hybrid trial including all cardiac rehabilitation centres that report to a national quality registry.
- The aim is to evaluate whether regularly reporting cardiac rehabilitation structure and process variables through a national quality registry and/or offering cardiac rehabilitation centres structured support to implement guidelines on secondary prevention will lead to an increase in centre-level adherence to guidelines on secondary prevention and improve patient-level outcomes.

## Background

Cardiovascular risk factor reduction and the fostering of a healthy lifestyle after an acute myocardial infarction (MI) are the most effective interventions to prevent recurrent coronary events [1]. Administering these interventions via structured and comprehensive cardiac rehabilitation (CR) programmes reduces mortality, morbidity, unplanned hospital admissions, and improves health-related quality of life [2–4]. CR is a complex intervention, combining the optimal use of cardio-protective medication, exercise training, patient education, and behavioural modification to improve lifestyle, and psychosocial counselling [5, 6]. Patient participation in CR after an MI is given the highest recommendation and level of evidence in current guidelines on cardiovascular disease (CVD) prevention [1]. However, referral rates to CR programmes are generally low and patients´ treatment target attainment is sub-optimal [7]. While treatment target attainment rates at Swedish CR centres is on average among the highest in Europe, there are considerable differences in CR service delivery between centres [8–11] and consequently, large variations in patient-level outcomes between centres [12].

The SWEDEHEART registry is a nationwide quality registry that records baseline characteristics, treatments, and outcomes of patients with MI admitted to coronary care units in Sweden [13]. Follow-up data describing secondary preventive patient-level outcomes have been collected in the CR part of the registry (SWEDEHEART-CR) since 2005 [14]. However, only a handful of variables monitoring centre-level structure and processes are included in the registry.

In 2019, a National Working Group on Secondary Prevention was commissioned by the Swedish Association of Local Authorities and Regions to author National Guidelines on Secondary Prevention for patients with coronary artery disease, aiming to decrease the variation in secondary prevention delivery and outcomes in Sweden. The guidelines were published in February 2022 [15]. In parallel with the release of CR guidelines, the SWEDEHEART-CR Working Group proposed incorporating variables into the registry to assess the recommended structure and processes outlined in the guidelines [16].

The Swedish healthcare system is highly decentralized, where overall healthcare policies and guidelines are set by national regulating agencies, and the responsibility for providing and funding services, lies with 21 autonomous regions [17]. Each regional authority is responsible for implementing the 2022 National Guidelines on Secondary Prevention on a local level. Implementing guidelines is, however, a complex process faced with many challenges, and often revised guidelines result in little or no change in clinical practice [18, 19]. To address the disparity between policies and clinical practice, the aims of the study are following:

*Primary aim*

- To prospectively study whether a) audit and feedback of CR structure and processes within the SWEDEHEART registry and b) supporting CR centres in implementing CR guidelines can increase centre-level guideline adherence.

*Secondary aims*

- To cross-sectionally evaluate the association between centre-level adherence to guidelines and patient-level outcomes.
- To prospectively study whether audit and feedback of CR structure and processes within the SWEDEHEART-CR registry can improve patient-level outcomes.
- To prospectively evaluate whether supporting CR centres in implementing CR guidelines can improve patient-level outcomes.
- To qualitatively evaluate barriers and facilitators to guideline implementation.
- To evaluate cost and cost-effectiveness of the implementation support.

## Method

### Study setting and recruitment

The study started in October 2023 and will include all CR centres (*n* = 78) which report to the SWEDEHEART-CR registry. The sole exclusion criterion for CR centres is unwillingness to participate. The inclusion criteria for patients are 1) having a diagnosis of a type 1 MI (caused by atherosclerotic plaque rupture or coronary artery thrombosis) and 2) age 18–79 years at discharge from MI hospitalization. There are no exclusion criteria for patients.

### Study design

The study is an open-label cluster-randomized effectiveness-implementation hybrid trial. The effectiveness-implementation hybrid design allows for testing an implementation strategy while observing the intervention´s impact on patient outcomes [20]. For the implementation support, the Consolidated Framework for Implementation Research (CFIR) model will be used to guide the design [21, 22]. Normalization Process Theory (NPT) will be used to guide the exploration of the implementation process [20].

### Randomization

Centres will be randomized 1:1:1 to three clusters (A, B and C). Randomization will be stratified by geographical healthcare district (two districts in each stratum). Randomization will be performed by an independent organisation not involved in the study to avoid site-selection bias.

### The interventions

**Fig. 1:**
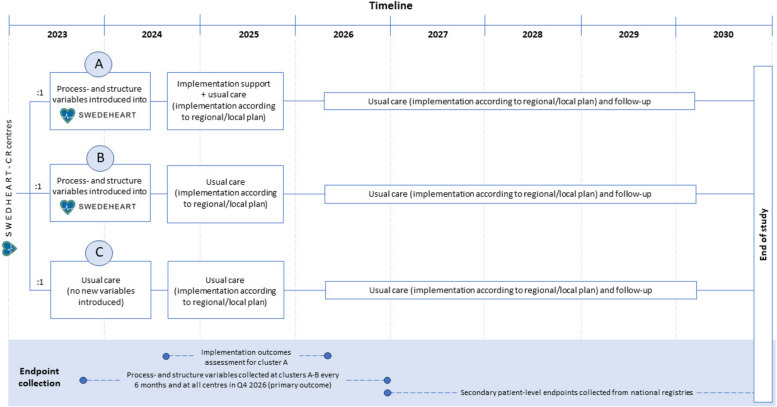
Overview, process and timeline of the study

First, baseline structure and process variables will be administered through the SWEDEHEART-CR registry at all CR centres. Second, two clusters will receive one of two interventions (A and B) and one cluster will receive no intervention (control cluster) (C) (Fig. 1):

A) Reprting CR structure and process variables through the registry every six months and being offered structured implementation support (audit intervention + implementation support intervention).
B) Reporting CR structure and process variables through the registry every six months but no structured implementation support being offered (audit intervention).
C) No reporting of CR structure and process variables, and no structured implementation support offered (control).

### The audit intervention

The audit intervention (clusters A and B) involves, on centre-level, reporting 30 variables on CR structure and processes, measuring adherence to the National Guidelines, to the SWEDEHEART registry. The complete list of CR structure and process variables is available in Additional file 1.

### The implementation support intervention

For the implementation support intervention (cluster A) facilitators from the research team will provide hands-on support and guidance to the CR team as they work to implement changes [23]. Facilitators are physicians and nurses experienced in the field of cardiac rehabilitation. In the recruitment step, the centre directors or key stakeholders will be contacted by the principal investigator to offer study participation by means of an e-mail. Upon acceptance to participate, the time to start the intervention will be determined, a timeline established, and the CR centre director will be asked to allocate time in the schedule for the relevant CR staff to work on the study. The implementation support will be conducted in four steps: 1) evaluation of centre practice, 2) identification of areas in need of improvement, 3) implementation of change, and 4) follow-up (Table 1). The implementation objects are work routines or CR programme components listed in Additional file 1 identified as sub-optimally implemented at the respective CR centre. Implementation strategies (defined by results from the Expert Recommendations for Implementing Change (ERIC) project) used will be identifying and preparing champions, identify barriers and facilitators, facilitation, distribution of educational materials, conducting educational meetings, organizing clinician implementation team meetings, and, providing local and technical assistance [23–28].

**Table 1:** The steps of the implementation support intervention

Steps of implementation support	Description of activities at each participating centre
Evaluation of centre practice	- The CR team (physicians, nurses, and physiotherapists) responds to a questionnaire listing aspects contributing to a best-practice CR model as defined in the National Guideline for Secondary Prevention [15] (Additional file 1). Using the responses current practice is evaluated the centre´s adherence to each object quantified on an ordinal scale (yes, partly, no). - Each member of the CR team responds to the Normalisation Measure Development (NoMAD) questionnaire [29].
Identification of areas in need of improvement	- Answers to the questionnaire listing aspects contributing to a best-practice CR are assessed by the facilitators from the research team and summarized in a report, also including feedback on possible areas of improvement. - The CR team receives a copy of the report. - Semi-structured focus group interviews performed on-site to identify barriers and facilitators to guideline implementation.
Implementation of change and run-in period	- An implementation is plan drafted. - Implementation assistance planned, prepared, and provided by the research team, with active participation of the healthcare personnel at the CR centre.
Study period	- Implemented changes followed for 4 months. Regular contact provided by the facilitators, assisting the centres in solving problems that can arise when adapting to new working routines.
Follow-up	- Each member of the CR team responds to the NoMAD questionnaire [29]. - Semi-structured focus group interview performed by the facilitators, to evaluate the implementation process.

The implementation support will be provided during a two-day visit from the facilitators, two follow-up phone calls during the first month after the visit, up to three educational meetings (number as requested by centre personnel), and a digital follow-up meeting 4 months after the initial visit. Additional phone or video calls, and emails will be provided as needed.

### Primary effectiveness outcome

The primary endpoint is an “adherence score”, measuring adherence to the National Guidelines on Secondary Prevention [15]. The adherence score is partly derived from the 30 variables capturing guideline-directed CR structure and processes incorporated into the SWEDEHEART-CR registry in October 2023. Permissible values are yes/partly/no/unknown. Additionally, the adherence score will include nine process variables that measure a) patient attendance in various CR components (number of patients attending a CR programme component divided by the number of patients eligible for participation), and b) time between MI hospitalization discharge and start of different CR programme components, already audited in SWEDEHEART:

- *Attendance in CR components*
  1. Proportion of patients attending an initial CR assessment (nurse visit).
  2. Proportion of patients attending an individual visit to a physiotherapist after discharge before starting an exercise-based CR (EBCR) programme (a pre-exercise screening visit).
  3. Proportion of patients completing a 3-month EBCR programme.
  4. Proportion of patients attending an individual close-out visit to a physiotherapist after completing an EBCR programme (a post-exercise assessment visit).
  5. Proportion of patients attending a patient education programme.
  6. Proportion of patients attending a close-out CR visit with a nurse at one-year after MI.
- *Time (days) to start of different components of CR*
  7. Time from hospital discharge to initial CR assessment (nurse visit).
  8. Time from hospital discharge to the pre-exercise screening visit to a physiotherapist.
  9. Time from pre-exercise screening visit to start of EBCR programme.

The responses will be pooled at each site into an adherence score for each CR centre. The contribution of each variable to the score will be weighed depending on the importance of each measurement, based on level of recommendation in European guidelines [1].

### Secondary effectiveness outcomes

Table 2 displays secondary outcomes, encompassing both short- and long-term patient outcomes and implementation outcomes. Implementation outcomes will be based on responses gathered through the customized Normalisation Measure Development (NoMAD) questionnaire [29] and insights obtained from focus group interviews, as well as a cost-effectiveness analysis.

**Table 2:** Secondary outcomes

Outcomes	Cluster	Measures	Data source	Timing
Short-term patient-level outcomes	All clusters	- Blood pressure (mmHg). - LDL-C (mmol/L). - Self-reported statue of health measured using EuroQol-VAS (0–10). - Self-reported smoking status (never/ prior [> 1 month smoke-free]/active) - Self-reported dietary habits (four questions with Likert scale 0–3 points, total score 0–12 points). - Self-reported physical activity (the number of days during the last week the patient has been physically active for a minimum of 30 min [at least 10 min at a time] with activity causing shortness of breath and a slightly increased pulse, corresponding to a brisk walk). - Completing an exercise-based CR programme for at least 3 months (yes/no)	The SWEDEHEART registry	Baseline and one-year after the start of intervention
		- Adherence to secondary prevention medication	Pharmaceutical registry	Baseline and one-year after the start of intervention
Long-term patient-level outcomes	All clusters	Total mortality Major adverse cardiovascular events (MACE) consisting of the following: - cardiovascular mortality - non-fatal MI - non-fatal ischemic stroke - coronary revascularization - hospitalization for new or worsening heart failure	National Patient Registry and the Cause of Death Registry	Up to 5 years after the end of the intervention
Centre-level implementation outcomes	Cluster A	- Barriers - Facilitators	Semi-structured focus group interviews	At the start and the end of implementation support period
		- Implementation cost and cost effectiveness.	A comparative health economic evaluation	At the start and the end of implementation support period

*LDL-C* low-density lipoprotein cholesterol, *VAS* visual analogue scale, *CR* cardiac rehabilitation, *MI* myocardial infarction

### Sample size calculations

As the number of CR centres is fixed, sample size calculations have been performed to estimate the difference in mean adherence score the study will be able to reveal. For this purpose, in a feasibility analysis the CR structure and process variables were collected from 8 CR centres of different sizes and geographical locations. The mean adherence score was 30.8 (standard deviation [± 2.4]) out of a maximum available score of 39. For the audit intervention, given the following presumptions:

- 23 out of possible 26 CR centres in each group (minimal drop-out is anticipated).
- Power of 80%.
- An alpha value of 0.05.

The study will have the power to identify a difference of ± 2.0 in adherence score between centres in cluster B (randomized to having variables on CR structure and processes incorporated in the SWEDEHEART registry) and cluster C (no new variables incorporated).

For the implementation support intervention, only centres in the lower 2 tertiles of the adherence score at baseline (approximately 17 centres) will be offered implementation support. Given the following presumptions:

- 14 out of possible 17 centres in each group (minimal drop-out is anticipated).
- Power of 80%.
- An alpha value of 0.05.

The study will have the power to identify a difference of ± 2.5 in adherence score between centres in cluster A (randomized to receiving implementation support) and cluster B (no implementation support).

### The timeline

The audit intervention started in October 2023 and will continue for 3 years. An interim analysis will be conducted two years after the start of the intervention. If the interim analysis shows the primary endpoint to be met (a difference of at least ± 2.0 in adherence score) the intervention will be terminated. Otherwise, the intervention will be continued until October 2026 and thereby uphold.

The implementation support intervention will start Q2 2024. Centres randomized to the implementation intervention will receive implementation support consecutively over a period of 18 months. The order in which centres will receive implementation support will depend on the centres’ possibilities and the research team´s capacity. The study outline is displayed in Table 3.

**Table 3:** Flow chart for the study

**Procedure**	2023		2024		2025		2026		2030
	Q3	Q4	Q1-2	Q3-4	Q1	Q3	Q1	Q3	
Randomization	√								
Structure and process variables - intervention sites (cluster A and B)	√	√	√	√	√	√	√	√	
Interim analysis						√			
Implementation support at intervention sites (cluster A)			√	√	√				
Utility of implementation assessment (cluster A)			√	√	√				
Structure and process variables -control sites (cluster C)	√					√		√	
End of audit intervention						(√)		√	
End of follow-up									√

### Data analysis

In the primary outcome analysis of the audit intervention, CR centres in clusters B and C will be compared. All CR centres that have responded to the CR structure and process variables will be included in the analysis. For the primary outcome analysis of the implementation support intervention CR centres in clusters A and B will be compared. All CR centres that have i) provided answers to the structure and process variables and that ii) have scores in the lower two tertiles of the adherence score at baseline will be compared. Our analyses will assume intention-to-treat principles by treating intervention assignment as randomized regardless of whether the intervention had uptake within the practice. If not all centres in cluster A accept implementation assistance, a per-protocol analysis will be performed including only those centres in study group A that accept implementation assistance.

For the secondary analyses on patient outcomes, all patients that attended at least two follow-up visits within CR will be included. For the outcome analysis of the audit intervention patients followed at CR centres in clusters B and C will be compared. For the outcome analysis of the implementation support intervention patients followed at CR centres in clusters A and B will be compared. A per-protocol analysis will be performed including only patients belonging to CR centres in cluster A that accept implementation assistance.

### Quantitative data

For baseline characteristics descriptive statistics will be used (means +/-standard deviation, medians [quartile 1, quartile 3], proportions [%] and ranges). For the primary outcome analysis, given randomized treatment assignment, total adherence scores at end of follow-up as well as change in scores between baseline and follow-up will be compared using linear regression analysis. In the case of unequal randomization concerning CR centre size (small < 75 patients/year, medium 75–150 patients/year or large > 150 patients per year), geography (6 geographical districts in Sweden, two in each stratum) or the centres belonging to a university hospital (yes/no), adjusted multivariable analysis will be performed. Results will be reported as relative treatment effects (odds ratios) with 95% confidence intervals. For secondary outcome analysis on short-term patient outcomes, the same statistical methods will be used, applying linear (continuous) or logistic (binary) regression analyses. For long-term outcomes (major adverse cardiovascular events [MACE] and total mortality) Cox proportional hazards regression models will be performed, reporting hazard ratios with 95% confidence intervals. In case of missing data, imputation will be considered. For all quantitative analyses a two-sided test of statistical significance will be used with an alpha level of 0.05.

### Qualitative data

For the implementation support intervention (cluster A), semi structured focus groups interviews will be conducted on each intervention site. A focus group will include 3–4 members of the CR team and a facilitator with experience of qualitative interviews will conduct the interviews. The interviews will assess the perception of contributing organizational, contextual, and structural factors that impact successful/unsuccessful uptake of the guidelines. Depending on number of centres accepting participation, approximately 10–12 interviews will be performed (pre- and post-implementation support intervention). Interviews will be conducted face-to-face or virtually via videoconferencing depending on the CR team´s and the facilitator´s availability and preference. To ensure anonymity, any identifiable information shared by participants will be dissociated from individual identities before analysis. The interview questions will be designed using the CFIR interview guide [30]. The interviews will be audio-recorded, transcribed verbatim, and analysed with descriptive, qualitative content analysis with an inductive and manifest approach according to Graneheim and Lundman [31, 32]. CFIR definitions and coding guidelines will be used to assist with coding of qualitative data [26].

### Cost and cost-effectiveness analysis

A comparative health economic evaluation of the implementation and the usual care models will be conducted. Generally, an economic evaluation serves to provide decision makers with relevant information about the value for money as to alternative treatment models. The economic evaluation will adopt generally accepted methods for such types of analyses, including the estimation of all relevant costs and benefits from both a health system and a societal perspective. Data on both direct and indirect costs of the two models will be collected through a survey where all centres will fill out the resources needed to ensure the implementation of the intervention.

Based on the cost estimates and the effect of the implementation assistance on patient outcomes, the economic evaluation will then be able to conduct a cost-effectiveness analysis (CEA). The CEA responds to the key policy question of the cost of the measured effects. Such information contributes to making informed priority decisions under fixed budget constraints in healthcare services. In addition, the *incremental* cost-effectiveness ratio (ICER) will be computed to assess the *added* costs relative to the *added* effects (benefits) of the intervention model compared with usual care:

$$\mathrm{ICER}=\mathrm{Cost{_-}t-Cost{_-}uc/Effects{_-}t-Effects{_-}uc}$$

where t = treatment option and uc = usual care option. The ICER shows the additional (incremental) costs of implementing the treatment model compared with the usual care option. Consequently, the ICER responds to the related policy question of how much more will be achieved for how much more resources (costs) compared with the current situation. The effect measures include those identified above under Study objectives (Sect. 4): guidelines adherence and patient-level outcomes.

### Ethical considerations and withdrawal criteria

The study will be performed in compliance with the study protocol, the Declaration of Helsinki, and current national and international regulations governing this clinical trial. The study has been approved by the Swedish Ethical Review Authority (Registration number: 2023-03217-01) and is registered at ClinicalTrials.gov (identifier: NCT05889416).

The SWEDEHEART registry is sanctioned by Swedish law, stating that all patients are informed of their inclusion and their right to opt-out and have their data erased at any time without a specific reason [13]. Opt-out is extremely rare, counting fewer than ten cases per year.

All centres report data to the SWEDEHEART registry on a voluntary basis. For the audit intervention an opt-out approach will be applied, i.e., CR centres not willing to provide answers to the new variables will be asked to convey this to the registry. Otherwise, if they submit answers to the new variables, they will be included in the analysis.

For the implementation support intervention, a perquisite for participation is a verbal consent from a centre director or key stakeholder followed by a signed Letter of Intent from the CR centre director.

### Data protection

The SWEDEHEART registry data is collected through an interactive web-based IT-platform, developed and maintained by Uppsala Clinical Research centres (UCR), Uppsala, Sweden. The data is electronically transferred to UCR in encrypted format and stored on a central server.

To ensure correct data matching and analysis, centre-level data (structure and process variables) will be requested in an identifiable form (i.e., name of CR centre is linked to data). All patient data from the SWEDEHEART registry and other national registries is, however, delivered pseudonymized to researchers, only containing a study identification number for each patient. As patient data contains information on at which centre the patients had their follow-up, matching to centre-level data will be done by CR centre. A patient-level identification key (study identification number linked to personal identification number) will be stored at the National Board of Health and Welfare, to allow for delivery of long-term outcome data – in the case of this study for up to 5 years.

All electronic study data delivered to the research team will be stored in a locked data storage requiring double identification for access. Only members of the research team will have access to data. Data processing will be performed in accordance with the provisions of the General Data Protection Regulation (GDPR) and other relevant legislation. No data will be shared outside of Sweden.

## Discussion

Referral rates to CR programmes after MI are generally low, and patients’ achievement of secondary preventive treatment targets is sub-optimal [7]. Despite well-defined frameworks for optimal CR in modern cardiology, there is considerable heterogeneity in the delivery of CR services across programmes and in patient outcomes [6, 9, 10, 12].

Quality registries offer unique possibilities to assess and compare the quality of care for patients. Monitoring the quality of healthcare is also crucial to reveal discrepancies between evidence-based recommended treatments and the actual care provided in clinical practice [33]. The audit and feedback process provided by registries has also been suggested to stimulate a question-behaviour effect among healthcare providers, even though scientific evidence is sparse [34, 35].

With the aim to decrease disparities in CR service delivery on a national level, Swedish Guidelines on Secondary Prevention were recently published. However, passively publishing guidelines is generally ineffective and leads to little change in clinical practice [19, 36]. If CR centres can optimize their delivery of services to meet standards set in guidelines, we may improve risk factor management and lifestyle, and decrease morbidity and mortality post-MI. This study has the potential to reveal barriers and facilitating factors that affect adoption to guidelines. Identifying barriers and facilitators opens opportunities to develop effective strategies and interventions to overcome them. This, in turn, may lead to a reduction in disparities between different centres promoting health equity.

### Strengths and limitations

A strength of our study is that patient-level data will be retrieved from coherent and consistent quality registries with national representability. On centre-level, the research team has a vast collegial network in Sweden, which is anticipated to aid in cultivating trust and engagement in the study. As for all implementation interventions, possible recruitment and retention barriers might be encountered, with possible lack of local leadership support and resistance to change in practice. At the same time, the facilitators will closely collaborate with CR teams to evaluate potential opportunities and obstacles. They will provide support and aim to establish trusting relationships with members of the teams. In the event of disruptions, their role will involve assisting the teams in effectively navigating and resolving these challenges.

### Impact

In addition to the potential impact on development of CVD across a large patient population, the work of implementing best practice to CR centres may engage local leaders in prioritizing CR and preventive patient care, increase CR centres awareness of their ability to change and adapt, and increase their collegial network within the field of preventive cardiology.

## Supplementary Information

Supplementary Material 1.

Supplementary Material 2.

Supplementary Material 3.

Supplementary Material 4.

Supplementary Material 5.

Supplementary Material 6.

## Data Availability

The datasets used and/or analysed during the current study are available from the PI on reasonable request.

## Abbreviations

CEA: Cost effectiveness analysis
CR: Cardiac rehabilitation
CVD: Cardiovascular disease
EBCR: Exercise-based cardiac rehabilitation
ERIC: Expert Recommendations for Implementing Change
EQ-VAS: EuroQoL Visual Analogue Scale
ICER: Incremental cost-effectiveness ratio
LDL-C: Low-density lipoprotein cholesterol
MACE: Major adverse coronary events
MI: Myocardial infarction
NoMAD: Normalisation Measure Development
NPT: Normalization Process Theory
Q: Quarter
SWEDEHEART: Swedish Web-system for Enhancement and Development of Evidence-based care in Heart Disease Evaluated According to Recommended Therapies
