# Supplementary material for: The effect of audit and feedback and implementation support on guideline adherence and patient outcomes in cardiac rehabilitation: a study protocol for an open-label cluster-randomized effectiveness-implementation hybrid trial
Source: Implement Sci. 2024 May 24;19:35. doi: 10.1186/s13012-024-01366-8 (PMC11531121; doi:10.1186/s13012-024-01366-8)
Supplement: Supplementary file 2 — Supplementary Material 2. [file 13012_2024_1366_MOESM2_ESM.docx]

Complete list of CR structure and process variables included in the SWEDEHEART-CR registry.

| Variable | Permissible values | | | |
| --- | --- | --- | --- | --- |
| ***Structure-based metrics*** | | | | |
| *Human resources* | | | | |
| The following professions are included in our CR team: | | | | |
| Nurse | Yes | Partly | No | Unknown |
| Physiotherapist | Yes | Partly | No | Unknown |
| Physician | Yes | Partly | No | Unknown |
| Social worker | Yes | Partly | No | Unknown |
| Psychologist | Yes | Partly | No | Unknown |
| Dietician/nutritionist | Yes | Partly | No | Unknown |
|  |  |  |  |  |
| The CR centre has a medical director with cardiology training who is responsible for the oversight of programme policies and medical procedures | Yes | Partly | No | Unknown |
| Nurses at the CR centre have an individual delegation to titrate dosage/suggest changes in lipid lowering therapy | Yes | Partly | No | Unknown |
| Nurses at the CR centre have an individual delegation to titrate dosage/suggest changes in blood pressure lowering therapy | Yes | Partly | No | Unknown |
| Personnel at the CR centre has training in counselling methods (e.g., motivational interviewing or cognitive behavioural therapy) | Yes | Partly | No | Unknown |
| At least one member of the CR team has training in tobacco counselling | Yes | Partly | No | Unknown |
| *Centre requirements* |  |  |  |  |
| We have regular interdisciplinary team meetings to discuss patient cases | Yes | Partly | No | Unknown |
| We have regular interdisciplinary team meetings to discuss operational matters such as work routines, quality of care, and to improve the team spirit | Yes | Partly | No | Unknown |
| We use SWEDEHEART-CR data continuously to follow and improve quality of care at our CR centre | Yes | Partly | No | Unknown |
| ***Process-based metrics*** | | | | |
| We follow and act on the patients´ identified modifiable risk factors | Yes | Partly | No | Unknown |
| We follow and act on the patients´ adherence to and effect of pharmacological treatment | Yes | Partly | No | Unknown |
| We strive for continuity in patient-caretaker contact throughout follow-up | Yes | Partly | No | Unknown |
| We offer the patients´ relatives to attend follow-up visits | Yes | Partly | No | Unknown |
| For non-Swedish speaking patients, certified interpreter services are used | Yes | Partly | No | Unknown |
| Nicotine-replacement therapy is offered to smokers | Yes | Partly | No | Unknown |
| Bupropion, cytisin or varenicline therapy is offered to smokers | Yes | Partly | No | Unknown |
| Assessment of alcohol consumption is included in routine follow-up | Yes | Partly | No | Unknown |
| We offer participation in supervised exercise-based CR for at least 3 months (24 sessions) | Yes | Partly | No | Unknown |
| For patients with high office blood pressure, we measure home and/or ambulatory blood pressure | Yes | Partly | No | Unknown |
| Fasting glucose and HbA1c are controlled during follow-up for all patients | Yes | Partly | No | Unknown |
| When fasting glucose and/or HbA1c are inconclusive, oral glucose tolerance test is performed | Yes | Partly | No | Unknown |
| Our cardiologists initiate and optimize treatment for type-2 diabetes | Yes | Partly | No | Unknown |
| Psychosocial status assessment and management (if needed) are included in routine follow-up | Yes | Partly | No | Unknown |
| Vocational counselling and support are included in routine follow-up | Yes | Partly | No | Unknown |
| We offer interactive patient education as a part of the CR programme | Yes | Partly | No | Unknown |

CR, cardiac rehabilitation; HbA1c, haemoglobin A1c; MI, myocardial infarction.
