# Supplementary material for: The effect of audit and feedback and implementation support on guideline adherence and patient outcomes in cardiac rehabilitation: a study protocol for an open-label cluster-randomized effectiveness-implementation hybrid trial
Source: Implement Sci. 2024 May 24;19:35. doi: 10.1186/s13012-024-01366-8 (PMC11531121; doi:10.1186/s13012-024-01366-8)
Supplement: Supplementary file 3 — Supplementary Material 3. [file 13012_2024_1366_MOESM3_ESM.pdf]

DECISION

2023-06-12

Study sponsor

Region Skåne

Primary investigator

Margrét Leosdottir

Title of project

The Effect of Audit and Feedback Within a National Registry and Implementation Support on Guideline Adherence and Patient Outcomes in Cardiac Rehabilitation

Information about the application

Application was received by the Swedish Ethical Review Authority 2023-05-22 and was validated 2023-05-26.

The Swedish Ethical Review Authority has decided on the application as stated below.

DECISION

The Swedish Ethical Review Authority approves of the research described in the application.

On behalf of The Swedish Ethical Review Authority

Åsa Ståhl

Chair

The decision has been made by the following persons:

Chair
Åsa Ståhl

Delegates with medical competence
Gunnar Göthberg (Pediatric surgery focusing on gastroenterology, pediatrics, surgery, research secretary)
Margareta Kreuter (Neurology, rehabilitation, sexuality, research secretary)
Bert Andersson (Cardiology, heart failure, representing delegate)
Elinor Bexe Lindskog (Colorectal cancer)
Anette Ekström-Bergström (Nursing science, reproductive health, perinatal and sexual health, gestational health, breastfeeding, health)
Charlotta Lundh (Clinical physiology)
Ulla Molander (Geriatrics)
Steinn Steingrimsson (Psychiatry, epidemiology, clinical trials)
Per Örtenwall (Surgery, vascular surgery)
Anna-Lena Östberg (Odontology, epidemiology, public health)

Delegates who represent public interests
Krister Andersson
Mona-Lisa Dahlberg
Jörgen Fransson
Ulla-Britt Hagström

The decision is sent to
The primary investigator: Margrét Leosdottir
Sponsor representative: Pia Malmkvist
