# Supplementary material for: The effect of audit and feedback and implementation support on guideline adherence and patient outcomes in cardiac rehabilitation: a study protocol for an open-label cluster-randomized effectiveness-implementation hybrid trial
Source: Implement Sci. 2024 May 24;19:35. doi: 10.1186/s13012-024-01366-8 (PMC11531121; doi:10.1186/s13012-024-01366-8)
Supplement: Supplementary file 4 — Supplementary Material 4. [file 13012_2024_1366_MOESM4_ESM.pdf]

Hjärt-Lungfonden

Beviljningsbrev för Projektbidrag (Hjärta)

Överläkare Margret Leosdottir
VO hjärt- och lungmedicin
Hjärtsjukvård Malmö
Skånes Universitetssjukhus
Jan Waldenströms gata 15 plan 3
20502 Malmö

Stockholm den 15 mars 2024

Bästa Margret Leosdottir,

Vi har glädjen att meddela dig att Hjärt-Lungfonden har beslutat att ge dig Projektbidrag (Hjärta) om 600 000 kronor för år 2020 , 600 000 kronor för år 2021, (nr. 20190431). Stödet har beviljats i hård konkurrens med andra forskningsansökningar. Hjärt-Lungfonden bedömer din forskning som mycket värdefull och har stora förhoppningar på resultat i framtiden. Det är med varm hand vi överräcker ditt anslag.

Vi vill öka insamlingen till stöd för fler viktiga forskningsprojekt. En viktig uppgift är därför att vi tillsammans berättar om alla de resultat som forskningen leder fram till och hur dessa resultat kommer våra givare och samhället i stort tillgodo. Det gör vi via många olika kommunikations- och insamlingsaktiviteter. När allmänhetens kunskap ökar blir insamlingarna mer framgångsrika och vi kan utöka utdelningen av forskningsstöd till dig och dina kollegor. Här har du som forskare en viktig roll! Läs mer i nedladdningsbar folder.

På Hjärt-Lungfondens forskningswebb har vi samlat all relevant information (www3.hjart-lungfonden.se). Där kan du läsa om dispositionstid, regler för utbetalning, ekonomisk och vetenskaplig redovisning samt om årsrapporter. Vid frågor om utbetalning av forskningsstöd ber vi dig ringa vår ekonomiavdelning, tel. 08-566 24 200 (vx).

Den indirekta kostnaden får maximalt utgå med 18 procent av beviljat belopp. För flerårigt anslag ber vi dig ange den indirekta kostnaden i samband med att du lämnar in din årsrapport. För forskartjänster och forskarmånader ingår den indirekta kostnaden i schablonbeloppet. Vad gäller resebidrag/kongressbidrag och utlandsstipendier dras inga indirekta kostnader. Hjärt-Lungfonden förbehåller sig rätten att låta oberoende revisorer göra stickprovskontroller av beviljade anslag och dess redovisning.

HJÄRT-LUNGFONDEN

Kristina Sparreljung
Generalsekreterare
