# Supplementary material for: The effect of audit and feedback and implementation support on guideline adherence and patient outcomes in cardiac rehabilitation: a study protocol for an open-label cluster-randomized effectiveness-implementation hybrid trial
Source: Implement Sci. 2024 May 24;19:35. doi: 10.1186/s13012-024-01366-8 (PMC11531121; doi:10.1186/s13012-024-01366-8)
Supplement: Supplementary file 5 — Supplementary Material 5. [file 13012_2024_1366_MOESM5_ESM.pdf]

FORTE
Forskningsrådet för hälsa, arbetsliv och välfärd

BESLUT
Beslutnr: STYA-2019/0004
Beslutsdatum: 2019-10-01

BESLUT OM BIDRAG

Diarienummer: 2019-00365
Projektledare: Margrét Leosdottir
Projekttitel: Effekter och kostnader gällande optimal hjärtrehabilitering för patienter efter hjärtinfarkt – Perfect CR studien

Beslutsinstans: STYA - Styrelsebeslut
Handläggare: Dag Hervieu

BESLUT
Forskningsrådet för hälsa, arbetsliv och välfärd har beslutat att ge bidrag enligt nedan:
2020-01-01 - 2020-12-31: 1 000 000 SEK
2021-01-01 - 2021-12-31: 1 000 000 SEK
2022-01-01 - 2022-12-31: 1 000 000 SEK

Beslutet tilldelas
Region Skåne
Projektledare: Margrét Leosdottir
